# Probabilistic fire spread forecast as a management tool in an operational setting

**DOI:** 10.1186/s40064-016-2842-9

**Published:** 2016-07-28

**Authors:** Renata M.S. Pinto, Akli Benali, Ana C. L. Sá, Paulo M. Fernandes, Pedro M. M. Soares, Rita M. Cardoso, Ricardo M. Trigo, José M. C. Pereira

**Affiliations:** 1 Centro de Estudos Florestais, Tapada da Ajuda, Instituto Superior de Agronomia, Universidade de Lisboa, Tapada da Ajuda, 1349-017 Lisbon, Portugal; 2 Centro de Investigação e de Tecnologias Agro-Ambientais e Biológicas, Universidade de Trás-os-Montes e Alto Douro (UTAD), Apartado 1013, 5001-801 Vila Real, Portugal; 3 Instituto Dom Luiz, Faculdade de Ciências da Universidade de Lisboa, 1749-016 Lisbon, Portugal

**Keywords:** FARSITE, Satellite active fires, MODIS, VIIRS, Uncertainty assessment and propagation, Fire growth modelling

## Abstract

**Background:**

An approach to predict fire growth in an operational setting, with the potential to be used as a decision-support tool for fire management, is described and evaluated. The operational use of fire behaviour models has mostly followed a deterministic approach, however, the uncertainty associated with model predictions needs to be quantified and included in wildfire planning and decision-making process during fire suppression activities. We use FARSITE to simulate the growth of a large wildfire. Probabilistic simulations of fire spread are performed, accounting for the uncertainty of some model inputs and parameters. Deterministic simulations were performed for comparison. We also assess the degree to which fire spread modelling and satellite active fire data can be combined, to forecast fire spread during large wildfires events.

**Results:**

Uncertainty was propagated through the FARSITE fire spread modelling system by randomly defining 100 different combinations of the independent input variables and parameters, and running the correspondent fire spread simulations in order to produce fire spread probability maps. Simulations were initialized with the reported ignition location and with satellite active fires. The probabilistic fire spread predictions show great potential to be used as a fire management tool in an operational setting, providing valuable information regarding the spatial–temporal distribution of burn probabilities. The advantage of probabilistic over deterministic simulations is clear when both are compared. Re-initializing simulations with satellite active fires did not improve simulations as expected.

**Conclusion:**

This information can be useful to anticipate the growth of wildfires through the landscape with an associated probability of occurrence. The additional information regarding when, where and with what probability the fire might be in the next few hours can ultimately help minimize the negative environmental, social and economic impacts of these fires.

**Electronic supplementary material:**

The online version of this article (doi:10.1186/s40064-016-2842-9) contains supplementary material, which is available to authorized users.

## Background

Portugal is a fire-prone country with one of the highest fire incidences in southern Europe (Ayanz et al. 2013). Landscape-level flammability was aggravated in the last four decades by the socio-economic and demographic trends that led to rural abandonment and consequent biomass accumulation (Costa et al. 2010; Marques et al. 2011; Fernandes et al. 2014). A growing number of studies using regional climate modelling have identified an increasing fire risk for the entire Iberia (Bedia et al. 2013; Sousa et al. 2015), particularly in Portugal (Carvalho et al. 2007; Pereira et al. 2013). According to future climatic scenarios, Portugal is expected to experience increasing temperatures in spring and summer and more frequent heat waves, likely leading to longer and more severe fire seasons (Ramos et al. 2011).

Catastrophic wildfires occurring under extreme weather conditions have already challenged the Portuguese fire suppression capabilities. During the 2003 fire season, extreme weather conditions were recorded with a devastating sequence of large wildfires resulting in around 450,000 ha of total burned area, approximately twice the previous highest record (220,000 ha in 1998) (Trigo et al. 2006). In 2005, as a consequence of one of the longest and most severe droughts of the last century, a total of 340,000 ha burned, making it the second worst fire year on record.

Spatially explicit fire spread models are an effective tool to study interactions between the main drivers of wildfire spread and behaviour—meteorological conditions, topography and vegetation (Keane et al. 2004), and have been widely used to simulate fire growth on the landscape (e.g. Keane et al. 2001; Stratton 2004; Loureiro et al. 2006; Arca et al. 2007; Cochrane et al. 2012). Although commonly used to study past fire events (hind-cast mode) they can also be used to predict fire spread during large wildfire events (forecast mode) in an operational setting. The predictions may assist in a better resource allocation, construction of fire control lines, and improved effectiveness of the initial attack (Calkin et al. 2011).

The operational use of fire spread models has largely followed a deterministic approach (Cruz and Alexander 2013), which does not account for predictions uncertainty. However, fire spread models are subject to assumptions and limitations that inherently produce compounding errors during simulations (Alexander and Cruz 2013; Cencerrado et al. 2014; Hilton et al. 2015). Furthermore, given our limited control over the quality of the model input data (Alexander and Cruz 2013; Bachmann and Allgöwer 2002), particularly in an operational setting, exact predictions of fire spread are difficult to achieve.

The uncertainty associated with model input variables and parameters needs to be acknowledged and tied to wildfire planning and decision-making (Thompson and Calkin 2011; Pacheco et al. 2015). Several works have integrated the uncertainty in fire growth modelling, using probabilistic approaches (Anderson et al. 2005, 2007; Carmel et al. 2009; Bar Massada et al. 2009; Cruz 2010; Calkin et al. 2011; Finney et al. 2011a, b; Hilton et al. 2015).

In alternative to fire spread modelling, some authors have also explored the potential of satellite active fire data to monitor large wildfire events (Englefield et al. 2004; Smith and Wooster 2005; Loboda and Csiszar 2007; Parks 2014; Veraverbeke et al. 2014). For example, data from the MODerate Resolution Imaging Spectroradiometer sensor (MODIS, Giglio et al. 2003) have been integrated in operational systems to assist fire managers (Frost and Annegarn 2007; Schroeder et al. 2008; Davies et al. 2009; Ressl et al. 2009).

Coen and Schroeder (2013) used satellite thermal data to initialize and evaluate coupled weather-wildfire growth model simulations, and obtained improved simulation results by using updated weather and fire location data. Anderson et al. (2007, 2009) also used active fires detected by MODIS and NOAA/AVHRR (National Oceanic and Atmospheric Administration Advanced Very High Resolution Radiometer) to build daily ignition grids.

Satellite active fire data and fire spread models provide different types of information regarding the spatial and temporal distribution of large wildfires. Our main goals are (1) to evaluate the potential of a probabilistic approach to integrate the uncertainty associated with some model inputs in fire spread simulations, using a spatially-explicit landscape fire spread model and (2) explore the potential of combining fire spread modelling with satellite active fires from consecutive MODIS and VIIRS (Visible Infrared Imaging Radiometer Suite, Justice et al. 2013) overpasses. By combining both tools we expect to attenuate some of the simulations errors, since the location of the fire front is updated at each overpass.

The feasibility of the proposed approach to forecast fire spread is examined based on the analysis of a well-documented large wildfire that occurred in southern Portugal in 2012 (Tavira wildfire, hereafter). We propose to (1) assess the probabilistic predictions of fire spread during the Tavira wildfire; (2) assess the combination of fire spread modelling and satellite active fire data; and (3) assess the decision-support potential of probabilistic fire spread to improve fire suppression in an operational setting, identifying if it could have been helpful to fire suppression and pre-suppression activities.

## Methods

### Case study: background and description

**Fig. 1:**
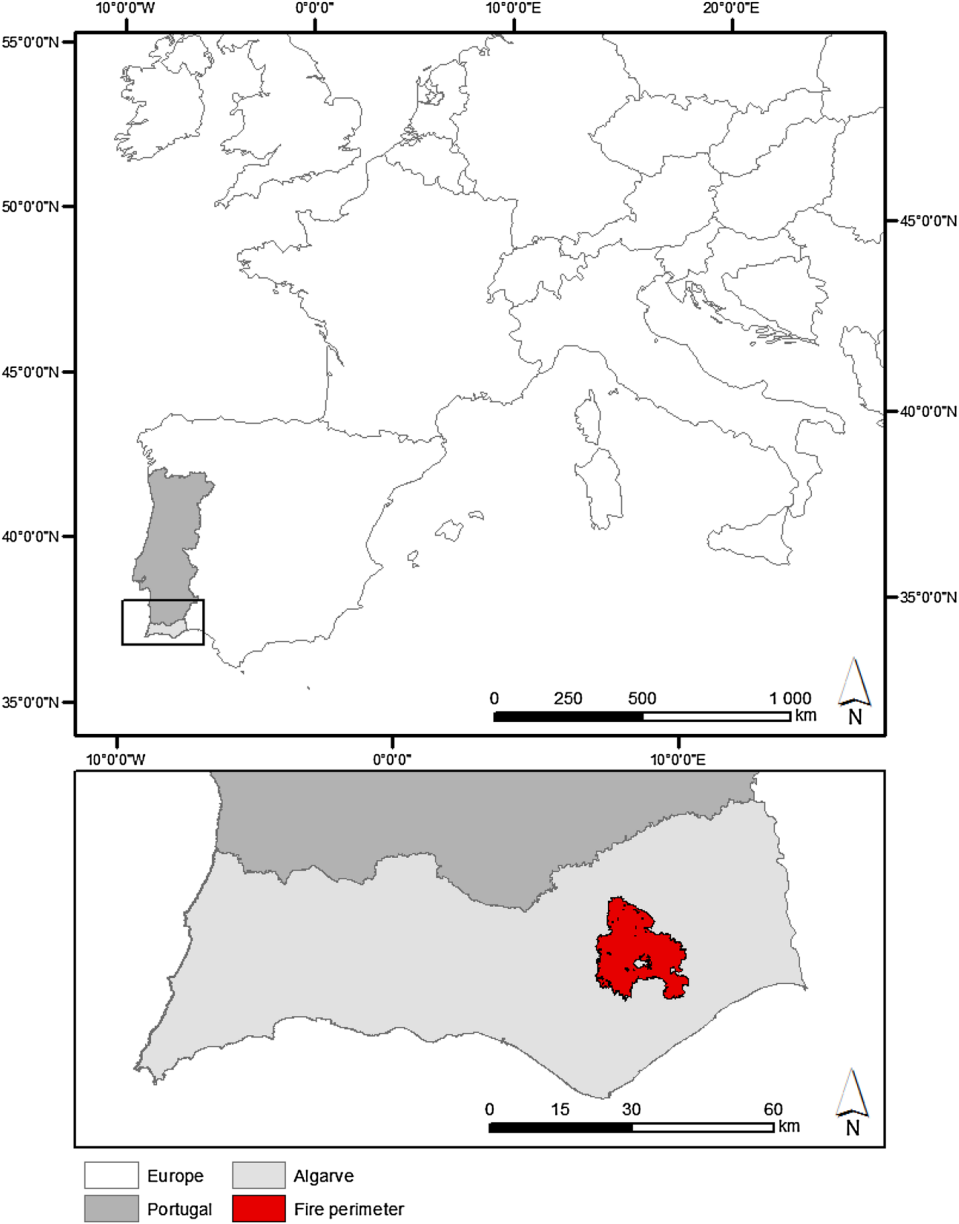
Location of Portugal in Europe and location of the Tavira wildfire in Portugal

The Tavira wildfire occurred between the 18th and 21st of July 2012 (ANPC 2012), in the Tavira and São Brás de Alportel municipalities, located in Algarve, southern Portugal (Fig. 1). It burned approximately 24,800 ha, mainly through shrublands (approximately 64 % of the landscape fire affected area; Additional file 1: Figure S1).

**Fig. 2:**
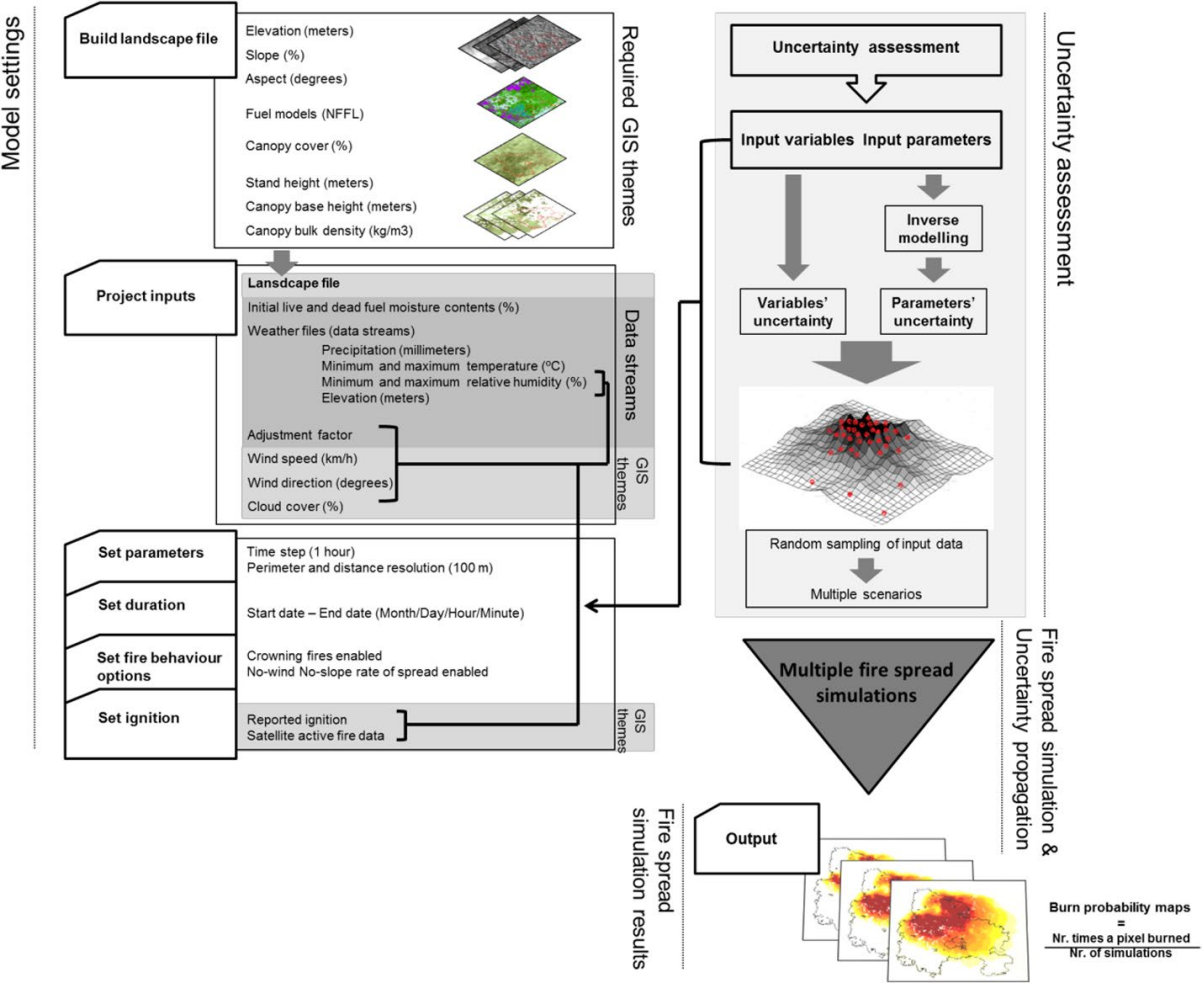
Diagram relating modelling stages, input variables, model settings and simulation results

**Fig. 3:**
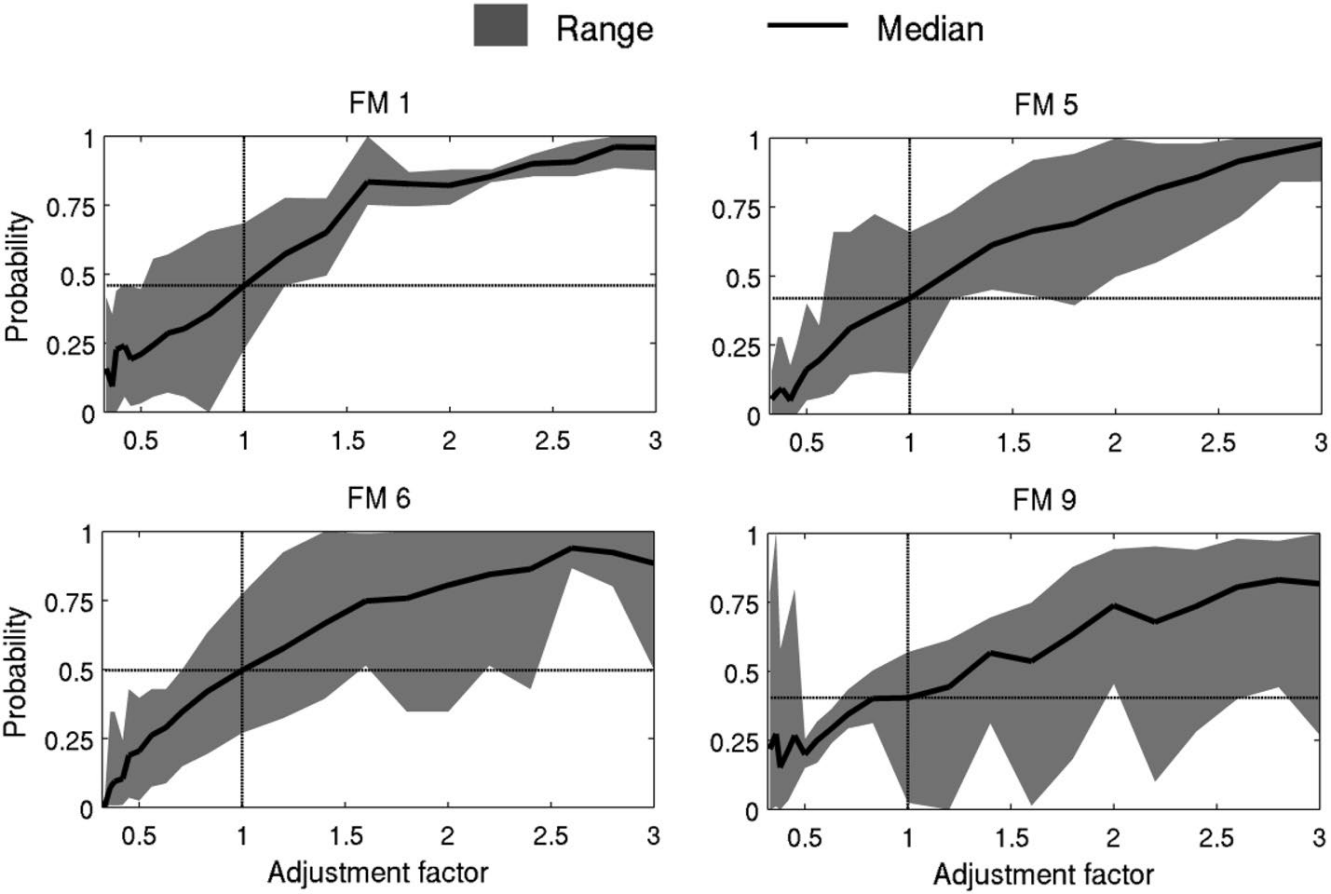
Estimated probability of the adjustment factors for NFFL fuel models 1, 5, 6 and 9. The range represents the minimum and maximum estimated probability when considering all eight wildfires

**Fig. 4:**
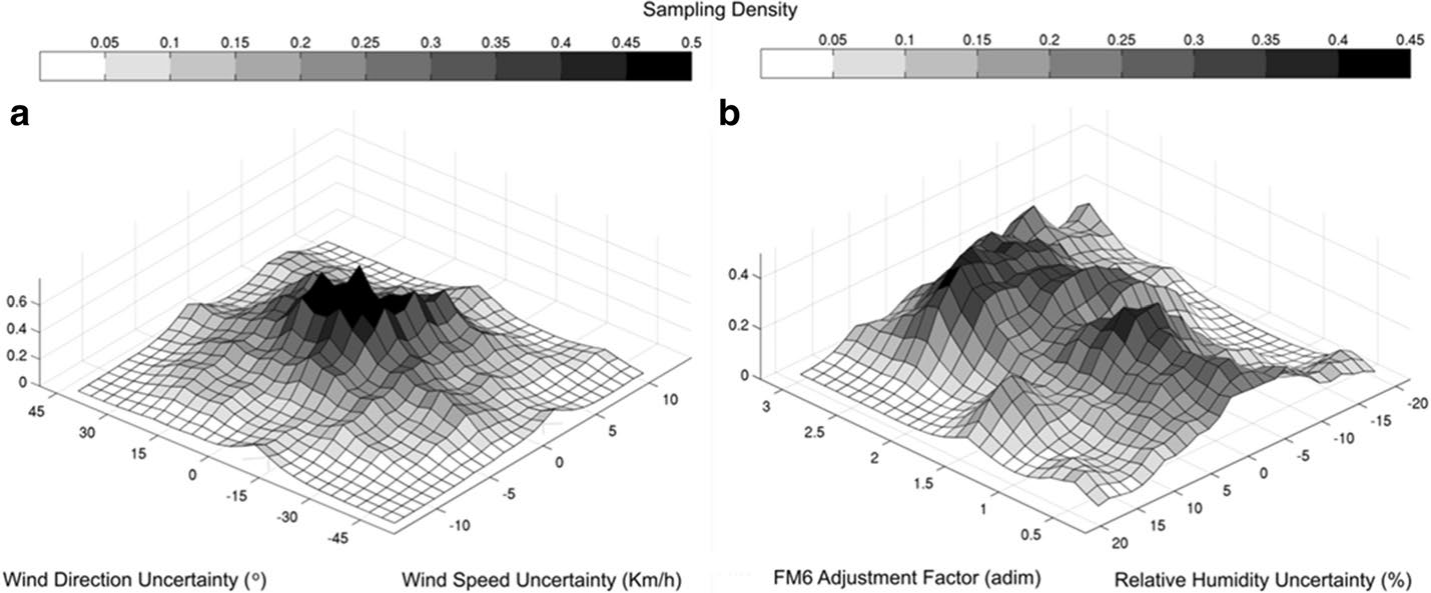
**a** Combined sampled point density of hourly wind direction and speed uncertainties; **b** combined sampled point density of daily relative humidity uncertainty and ROS adjustment factor for fuel model 6

The fire spread through heterogeneous terrain, with prevailing steep slopes (>20 %) and higher altitude in the north region of the Tavira municipality. Less steep terrain (0–20 %) and lower altitudes are found at the southeast area of Tavira and southwest area of the São Brás de Alportel municipalities, with plains at several locations (Viegas et al. 2012) (Additional file 1: Figure S2). The climate is Mediterranean, with the annual average temperature ranging from 10 to 25 °C, and average maximum temperature values ranging from 22 °C to more than 30 °C in August, with maximum absolute temperatures around 39 °C. During summer, relative humidity registers mean values below 65 % (ANPC 2012).

It should be stressed that in 2012 climatic conditions observed in Iberia were significantly different from the average, having experienced an extreme drought (Trigo et al. 2013). In particular, precipitation in Tavira was 45 % below the normal record and the entire study area was under extreme drought condition, with a soil water content value below 10 % at the time of the fire (Viegas et al. 2012). In addition, the years of 2010 and 2011 experienced above average precipitation, thus favouring vegetation growth and fuel build up. Fire danger as per the Canadian Fire Weather Index (FWI) System was rated *Extreme* with FWI = 56.7 during the most active fire spread period (Viegas et al. 2012).

The Tavira wildfire was first reported on July 18 (at ≈13 h UTC), contained on July 21 (at ≈17 h UTC) and extinguished on July 27. Two important stages for the wildfire event analysis were identified in the reports and will be briefly described (ANPC 2012, Viegas et al. 2012).

#### First stage: initial development, from 13 h July 18–17 h July 19 (approx. 28 h)

**Fig. 5:**
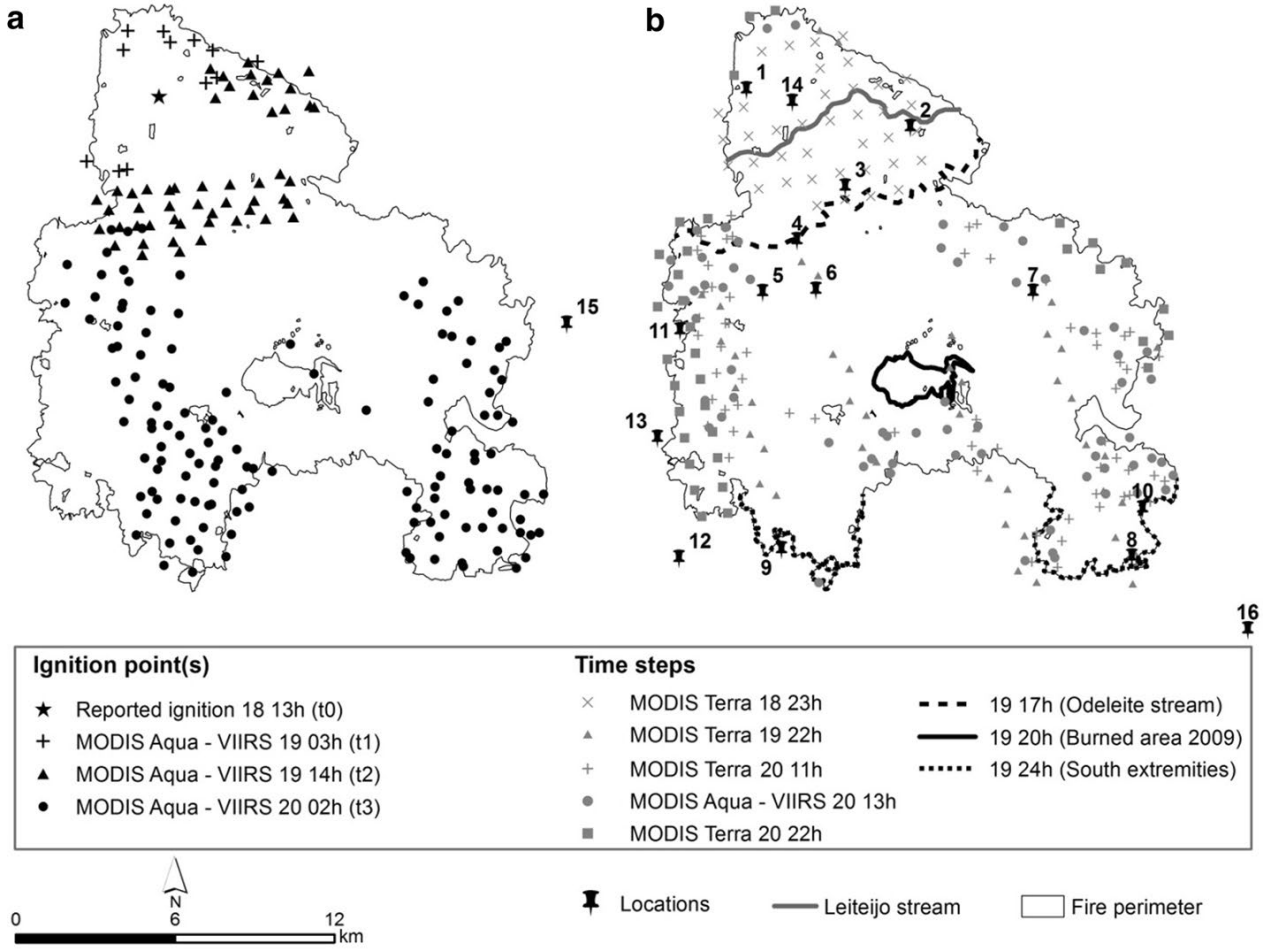
Ignition points and time-steps used in the simulations’ framework, based on reported and satellite information. **a** Ignition point(s) used to initialize simulations (day and time); **b** spatial and temporal information obtained from the Tavira wildfire reports and satellite active fires (day and time). See Table 1 for a more detailed description of the wildfire development and reference locations

The fire burned approximately 5000 ha (about 20 % of total burned area) during this stage, under favourable conditions for fire spread. Fuel moisture was low, allowing for ember projection and ignition up to hundreds of meters, resulting in multiple spot fires. Wind direction was highly variable, causing frequent shifts in the direction of maximum spread (making the initial attack difficult), which was mainly to south/southeast until it reached the Leiteijo stream (Fig. 5b; section “Probabilistic fire spread simulations: framework and assessment”), where it increased speed under the influence of topography. Around 16:30UTC on July 19, the fire started spreading through steep slopes along the Odeleite Stream (Fig. 5b; section “Probabilistic fire spread simulations: framework and assessment”). Fire spread fast until 17 h July 19 with few opportunities for direct attack (operations were mainly focused on the protection of lives and properties) and fire suppression was hampered by the unfavourable steep, rugged terrain. At this stage, the fire burned approximately 5000 ha in 28 h (≈180 ha/h).

#### Second stage: conflagration with very fast spread towards south, July 19 from 17 h to 24 h (approx. 7 h)

The fire reached the Odeleite Stream and turned into a major conflagration, making its suppression extremely difficult. Several factors led to this loss of control. Upon reaching the Odeleite Stream the fire increased in intensity when its spread became orographically channelled. Moreover, an increase in wind speed led to fast and intense fire growth towards south through areas with high fuel loading, and with a 10 km fire front split into two advanced sections heading west and east to the São Brás de Alportel (Fig. 5b, location 12; section “Probabilistic fire spread simulations: framework and assessment”) and the Tavira (Fig. 5b, location 16; section “Probabilistic fire spread simulations: framework and assessment”) municipalities, respectively. Spotting occurred up to two kilometres ahead of the fire front. Fire spread between this stage and the southern limit of the fire perimeter (ICNF 2012) in approximately 7 h (Fig. 5b; section “Probabilistic fire spread simulations: framework and assessment”). At this stage the fire burned approximately 20,000 ha in 7 h (≈2860 ha/h), 15 times faster than the previous period.

### Fire spread model and input data

FARSITE (Fire Area Simulator, Finney 2004) is a two-dimensional deterministic fire growth and behaviour model, developed by the USDA Forest Service, which integrates a surface fire spread model (Rothermel 1972) with models for crown fire transition (Wagner 1977) and crown fire spread (Rothermel 1991). The spatial growth of fire perimeters is based on Huygens’ Principle of wave propagation (Finney 2004).

Spatial inputs required for modelling an individual fire are topography, fuel models, canopy cover, stand height, canopy base height and canopy bulk density, wind speed and direction (hourly sampling), and ignition location. Additionally, FARSITE requires data streams of daily weather observations of minimum and maximum temperature, minimum and maximum relative humidity, precipitation and cloud cover at a specified elevation, and initial live and dead fuel moisture contents (Fig. 2, section “Uncertainty assessment and propagation”).

Elevation was obtained from the Digital Elevation Model provided by the Shuttle Radar Topography Mission, at 90 m spatial resolution (Farr et al. 2007). The dataset was also used to derive the slope and aspect variables. Fuel maps were sourced from the Portuguese municipalities affected by the wildfire, adopting the Northern Forest Fire Laboratory (NFFL) 13 standard fire behaviour fuel models (Anderson 1982). Canopy cover (%) was derived from the MODIS Vegetation Continuous Fields Yearly L3 Global 250 m (MOD44B) tree cover dataset (DiMiceli et al. 2011). Stand height, canopy base height and canopy bulk density data were acquired from the Portuguese National Forest Inventory (2005–2006) (DGRF 2006) (Additional file 1: Table S1).

Wind and the other meteorological variables were derived from a high, vertical and horizontal, resolution regional climate simulation, performed with the Weather Research and Forecast model, version 3.1.1 (WRF3.1.1, Skamarock et al. 2008). In this simulation the ERA-Interim reanalysis (Dee et al. 2011) is dynamically downscaled for the Iberian Peninsula, at 9 km resolution, over the period 1989–2013 (Soares et al. 2012). The output sampling is hourly for all variables, and the simulation quality was already extensively evaluated for temperature (Soares et al. 2012), precipitation (Soares et al. 2012; Cardoso et al. 2013), wind (Soares et al. 2014) and solar radiation (Magarreiro et al. 2015). The weather data used in this study has the same resolution as the weather forecasts produced by IPMA (Portuguese Sea and Atmosphere Institute) using the Aladin model (9 km horizontal resolution with a 72 h forecast range, Yessad 2011), and provided to the National Authority for Civil Protection (ANPC) during fire operational settings.

WindNinja (version 2.1.3, Forthofer 2007) was used to spatially model the prevailing hourly wind inputs, given the interaction with topography (gridded input). Temperature, relative humidity and precipitation were summarized on a daily basis (data stream input), given the time of maximum and minimum temperature. Cloud cover was assumed to be zero for the entire simulation period (see Table 1 for a summary of the datasets used to derive the model input data).

**Table 1:** Tavira wildfire development and behaviour description based on reported information

Ignition point(s)	Time steps	Wildfire development	Additional remarks
18 13 h (t_0_) ٭ 19 03 h (t_1_) + 19 14 h (t_2_) ▲	18 23 h ×	Stage 1 (≈28 h): wind directions highly variable, causing the fire to constantly shift its head-fire section, growing mainly to south/south-east until it reached Leiteijo stream, increasing its speed. Day 18 at ≈15 h the fire reached location 1. Day 18 at ≈23 h the fire had 4 active fronts, two fronts standing out—one heading east towards location 2, and the other heading south towards locations 3 and 4	Two fire occurrences in a contiguous municipality, day 19 ≈13 h and 14 h were reported. All areal resources that were slowing fire progression towards south were displaced to contain these fires
	19 17 h ╍	Stage 2 (≈7 h): fire reached Odeleite Stream and turned into a major conflagration. Wind speed increased and led to a fast and intense growth towards south with a 10 km fire front and two advanced sections, one heading west towards locations 5 and 6 and the other heading east to location 7. Around 18h30 m the fire was close to location 7. In approximately 3-5 h the fire burned an area greater than the previously consumed in 28 h, traveling a distance of ≈6 km in the west part and 7.5 km in the southeast part. The fire reached its extremities in approximately 7 h	Fire reaches Odeleite Stream
	19 20 h ━		Fire reaches the area burned in 2009
	19 22 h △		
	19 24 h ┅		Fire reaches its south limit
20 02 h (t_3_) ●	20 11 h + 20 13 h ○ 20 22 h □	Day 20 at ≈2 h the fire was reported at location 8, 15 km distance from the beginning of stage 2 (2 km/h). At ≈2 h 30, the fire front from the west side was heading in direction of location 9. This fire front was being contained with bulldozers, areal and terrestrial resources. Fire was globally controlled in the other fronts. More resources became available from the other fire occurrence. By the end of the day, the highest fire activity was observed at locations 10 and 11, with the remaining sectors consolidated or with favourable evolution. Meteorological conditions became less severe during the night (higher relative humidity and lower temperature)	Day 20 at ≈6 h the fire in the contiguous municipality was controlled
		Day 21 at ≈6 h, bulldozers started working between locations 12 and 13, and locations 14 and 13 (fire control line with 14 km length and 20 m wide, in 48 h) preventing fire spreading to location 15, increasing its severity to great extent	Wind direction change forecast to north-west, with possible spread towards location 15

Ignition point(s) and time-steps used in the simulations framework and assessment. For symbols see Fig. 5

A standard fuel moisture scenario (Scott and Burgan 2005) was used as reference for live and dead fuel moisture content values. Dead 1-, 10- and 100-h fuel moisture content values were set to 6, 7 and 8 %, respectively. Live herbaceous and woody fuel moisture content values were set to 60 and 90 %, respectively (scenario D2L2—low moisture content).

Since a large number of simulations were performed, FARSITE 4 command line version was used. Landscape spatial resolution, perimeter and distance resolution, and temporal resolution were set to 100 m cell-size and 1 h time step, respectively. Fire suppression activities and spotting were not simulated. Crowning fires and no-wind no-slope rate of spread for the spread rate of backing fires were enabled during simulations.

### Satellite active fire data

Satellite data provides relevant information regarding the spatial and temporal fire spread dynamics of large wildfire events (Veraverbeke et al. 2014; Parks 2014). Active fire data from two satellite sensors were used to re-initialize fire spread simulations, using active fires as ignition points (Anderson et al. 2009) to update the fire location at each satellite overpass (Additional file 1: Table S1).

The MODIS active fire product (MCD14ML) detects fires in 1 km pixels that are burning at the time of overpass under relatively cloud-free conditions up to four times per day (Giglio et al. 2003). Day and night time overpasses from both satellites, Terra (≈10:30 h and ≈22:30 h UTC) and Aqua (≈14 h and ≈2 h UTC) are included in the MODIS active fire product. Each overpass has a different viewing geometry and thus a different pixel size (Wolfe et al. 1998). The active fire pixel size was defined based on the viewing zenith angle and on the azimuth of each MODIS overpass (Ichoku and Kaufman 2005).

The VIIRS Active Fires product has similar overpass times as MODIS Aqua, crossing the equator two times a day at 1:30 pm (ascending node) and at 1:30 AM (descending node), with improved spatial resolution (750 m) (Justice et al. 2013). The VIIRS sensor operates with a specific pixel aggregation scheme, with the along-scan growth of pixel size significantly reduced. In addition, VIIRS is expected to detect more fires than MODIS, given its spatial resolution (Csiszar et al. 2014). Both active fires products were used: (1) to verify data consistency; (2) have more detailed information of the spatial–temporal distribution of fire growth.

### Uncertainty assessment and propagation

The reliability of FARSITE simulations partly depends on the accuracy of input data (Finney and Ryan 1995; Alexander and Cruz 2013; Cruz and Alexander 2013; Duff et al. 2013; Cencerrado et al. 2014). Usually, the only readily available input data with acceptable accuracy are the topographic variables slope, aspect, and elevation. However, the required weather data and additional parameters, such as the rate of spread (ROS) adjustment factors, are often difficult to obtain, define or validate, especially in a moment of emergency (Cruz and Alexander 2013; Cencerrado et al. 2014). The use of fire spread models, particularly as a decision-support tool, should quantify the uncertainty of model predictions (Mowrer 2000; Thompson and Calkin 2011). Mowrer (2000) and Bachmann and Allgöwer (2002) have propagated uncertainty in fire spread modelling through the contributions of the input variables and parameters’ uncertainties.

Several variables affect the variability of fire spread modelling outputs, such as wind speed (Anderson et al. 2005, 2007; Cruz 2010; Finney et al. 2011a; Hilton et al. 2015), wind direction (Anderson et al. 2005; Finney et al. 2011a; Hilton et al. 2015), relative humidity (Anderson et al. 2005, 2007; Cruz 2010), ignition location (Bar Massada et al. 2009) and ROS adjustment factor parameter. The uncertainty associated with these variables results from their natural variability and the unfeasibility to accurately forecast them. Regarding satellite active fire data other factors may constrain the sensor detection rate, for example the occurrence of persistent cloud clover and/or dense smoke plumes (Csiszar et al. 2006; Giglio et al. 2003; Hantson et al. 2013).

For relative humidity, uncertainty was quantified by comparing minimum and maximum daily relative humidity model estimates with measurements done in meteorological stations over Portugal for the summer period (Benali et al. 2016). According to these authors, the resulting probability density functions (PDF) for both variables were similar, thus we decided to define a single uncertainty PDF. For wind speed and direction, uncertainty was quantified by the authors using a multi-model approach (Refsgaard et al. 2007). Based on the previous work, we defined empirical uncertainty PDF for relative humidity and wind speed and direction, described by a normal distribution with $\bar{x}$ = 0 % and σ = 8.5 %, $\bar{x}$ = 0 km/h and σ = 5.5 km/h and $\bar{x}$ = 0º and σ = 20º, respectively. Cruz (2010) also used Gaussian PDFs to describe the uncertainty associated with relative humidity, air temperature and wind-related variables.

The uncertainty associated with satellite active fire location was also defined empirically taking into account the pixel’s geometry (Wolfe et al. 1998; Justice et al. 2013; Campagnolo and Montano 2014). Regarding the active fire data we assumed the centroid coordinate and assigned the closest 100 m grid cell. To account for uncertainty in sub-pixel fire front location and pixel footprint size variability, we randomly sampled points within 1500 and 500 m of centroid coordinates of MODIS and VIIRS active fires, respectively. For reported ignition location(s) we assumed a 250 m radius, to account for location uncertainty.

The ROS adjustment factor is a FARSITE parameter commonly used by fire managers to adjust the outputs based on expert knowledge of the expected fire behaviour for each fuel model (FM) class. Since no data is available regarding the distribution of the ROS adjustment factor, we used eight wildfires that occurred in Portugal between 2003 and 2005 to estimate the uncertainty PDFs for the ROS adjustment factor parameter through an inverse modelling approach (Refsgaard et al. 2007). The impact of uncertainty of this parameter was assessed given the observed discrepancy between simulated fire spread and satellite thermal active fires, for eight wildfires (data not shown). Each adjustment factor was varied between 0.33 and 3 for each fuel model class independently, corresponding to a threefold decrease or increase in fire spread rate, respectively. The uncertainty PDF was assumed to be inverse to the simulated-satellite spatial discrepancy, i.e. for a given adjustment factor value, the lower the spatial discrepancy, the higher the probability of the adjustment factor being correct. The uncertainty adjustment factors PDF was derived for the most representative fuel models of the eight case studies. The estimated uncertainty PDFs for NFFL fuel models 1 (grass), 5 and 6 (shrub) and 9 (litter) are shown in Fig. 3.

For relative humidity and wind speed and direction, the values were sampled from the uncertainty PDFs. For the ROS adjustment factors, we sampled values from the uncertainty PDFs estimated for FM classes 1, 6 and 9 (covering 13.0, 40.4 and 9.8 % of the study area, respectively). For the remaining classes (FM2—10.3 %, FM4—1.3 %, FM5—19.2 %, FM7—4.7 % and FM8—1.4 %) for which uncertainty PDFs were not estimated previously, the adjustment factors were randomly sampled between 0.33 and 3. For active fires and ignition location, values were randomly sampled within the radii defined above.

Figure  4a shows the combination of sampled uncertainty values for hourly wind direction and wind speed. For example, when uncertainty had a positive signal, the sampled value was added to the reference value, and vice versa, i.e. when a 10 km/h wind speed uncertainty value was sampled, this value was added to the input hourly wind streams. Since uncertainty for both variables was defined considering normal distributions centred on 0, the combination is a bell-shaped probability surface. Figure 4b shows an example of the combination between sampled ROS adjustment factor (for fuel model 6) and sampled uncertainty of daily relative humidity. The surface is quite different from the one shown in Fig. 4a, since the adjustment factor PDF presents a bimodal configuration with two distinct peaks centred in values close to 1 and between 2 and 3.

Finally, uncertainty was propagated through the FARSITE fire spread modelling system by randomly defining 100 different combinations of the independent input variables, and running the correspondent fire spread simulations.

### Probabilistic fire spread simulations: framework and assessment

Multiple fire spread simulations were performed accounting for the uncertainty in specific model inputs, as described in the previous section. To produce the fire spread probability maps, we run FARSITE 100 times for each set of simulations varying simultaneously: (1) relative humidity; (2) wind speed and direction; (3) the ignition location; and (4) the ROS adjustment factors. Multiple interactions between these variables produce several possible fire spread predictions, creating a probabilistic representation of fire growth. FARSITE outputs include fire perimeters and several fire behaviour parameters. The fire’s Time of Arrival (TOA, in hours) was used to create the burn probability maps, using all TOA outputs from each set of simulations. The burn probability was calculated as the ratio between the number of times a given pixel burned and the total number of simulations, expressed in percentage.

The simulation framework was designed to emphasize the most active period of the Tavira wildfire: from ignition (day 18 at 13 h) until it reached its southern edges (day 19 at 24 h) (UTC time). Simulations were initialized at the reported ignition location, day 18 at 13 (t_0_), and re-initialized with active fires from combined overpasses of both MODIS Aqua and VIIRS satellites, day 19 at 3 h (t_1_), day 19 at 14 h (t_2_) and day 20 at 2 h (t_3_) (Fig. 5a, Table 1). Different time steps were defined to establish the duration of each simulation and compare the probabilistic results with the spatial–temporal distribution of active fires and the reported fire front location information.

Based on the documented Tavira wildfire spread and behaviour, three key time steps (burning periods) were defined: day 19 at 17, 20 and 24 h (Fig. 5b, Table 1). Additional time steps were defined for further comparison, based on MODIS Terra, MODIS Aqua and VIIRS satellite overpasses, day 18 at 23 h (MODIS Terra), day 19 at 22 h (MODIS Terra), and day 20 at 11 h (MODIS Terra) and at 13 h (MODIS Aqua—VIIRS); the last time step, day 20 at 22 h (MODIS Terra) represents the time when the Tavira wildfire reached its eastern and western boundaries (Fig. 5b, Table 1).

**Fig. 6:**
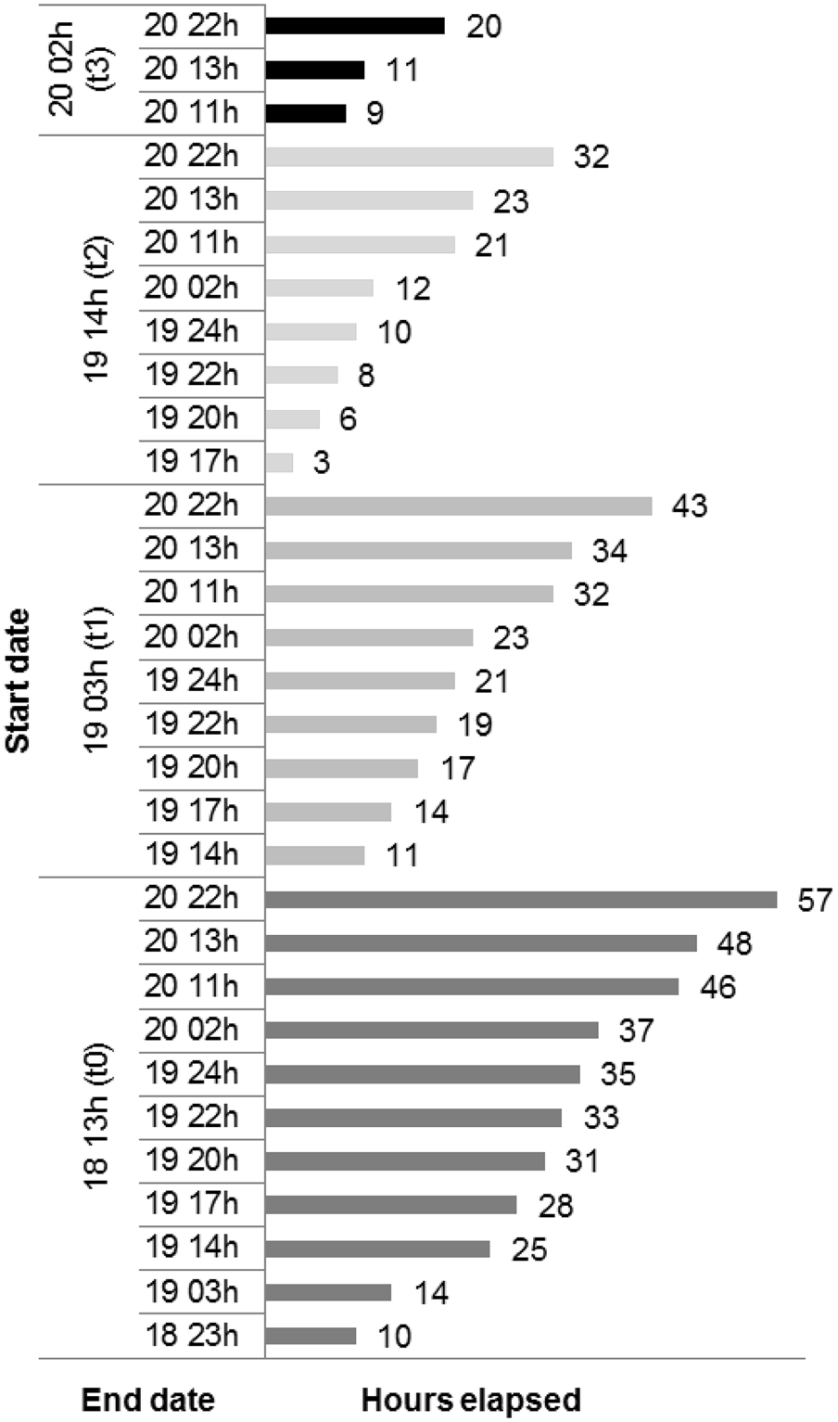
Simulations duration framework. Simulations ran sequentially from the ignition point(s) t_0_, t_1_, t_2_, t_3_ (start date) and between the defined time steps (end date)

A total of 31 simulations sets (100 runs each) with different durations were performed (Fig. 6): simulations *1 to 11*—initialized at the reported ignition point (start: day 18 at 13 h (t_0_); durations: 10, 14, 25, 28, 31, 33, 35, 37, 46, 48 and 57 h, respectively); simulations *12 to 20* - initialized with satellite active fires ignition points from the MODIS Aqua - VIIRS overpasses (start: day 19 at 3 h (t_1_); durations: 11, 14, 17, 19, 21, 23, 32, 34 and 43 h, respectively); simulations *21 to 28*—initialized with satellite active fires ignition points from the MODIS Aqua—VIIRS overpasses (start: day 19 at 14 h (t_2_); durations: 3, 6, 8, 10, 12, 21, 23 and 32 h, respectively) and simulations *29 to 31*—initialized with satellite active fires ignition points from the MODIS Aqua -VIIRS overpasses (start: day 20 at 2 h (t_3_); durations: 9, 10 and 20 h, respectively). For comparison, deterministic simulations were also performed, initialized with the same ignition points and with equal start and end dates.

To evaluate fire spread predictions, the burn probabilities were temporally and spatially compared with two independent sources of information: the commissioned Tavira wildfire reports (ANPC 2012; Viegas et al. 2012) and satellite active fires from MODIS and VIIRS sensors. Regarding the latter, for each satellite active fire, the maximum simulated fire spread probability within its footprint was recorded. The median simulated probability was calculated for all the active fires acquired at a given time step, to compare the fire spread probability maps with satellite information.

## Results and discussion

### Probabilistic predictions of fire spread

**Fig. 7:**
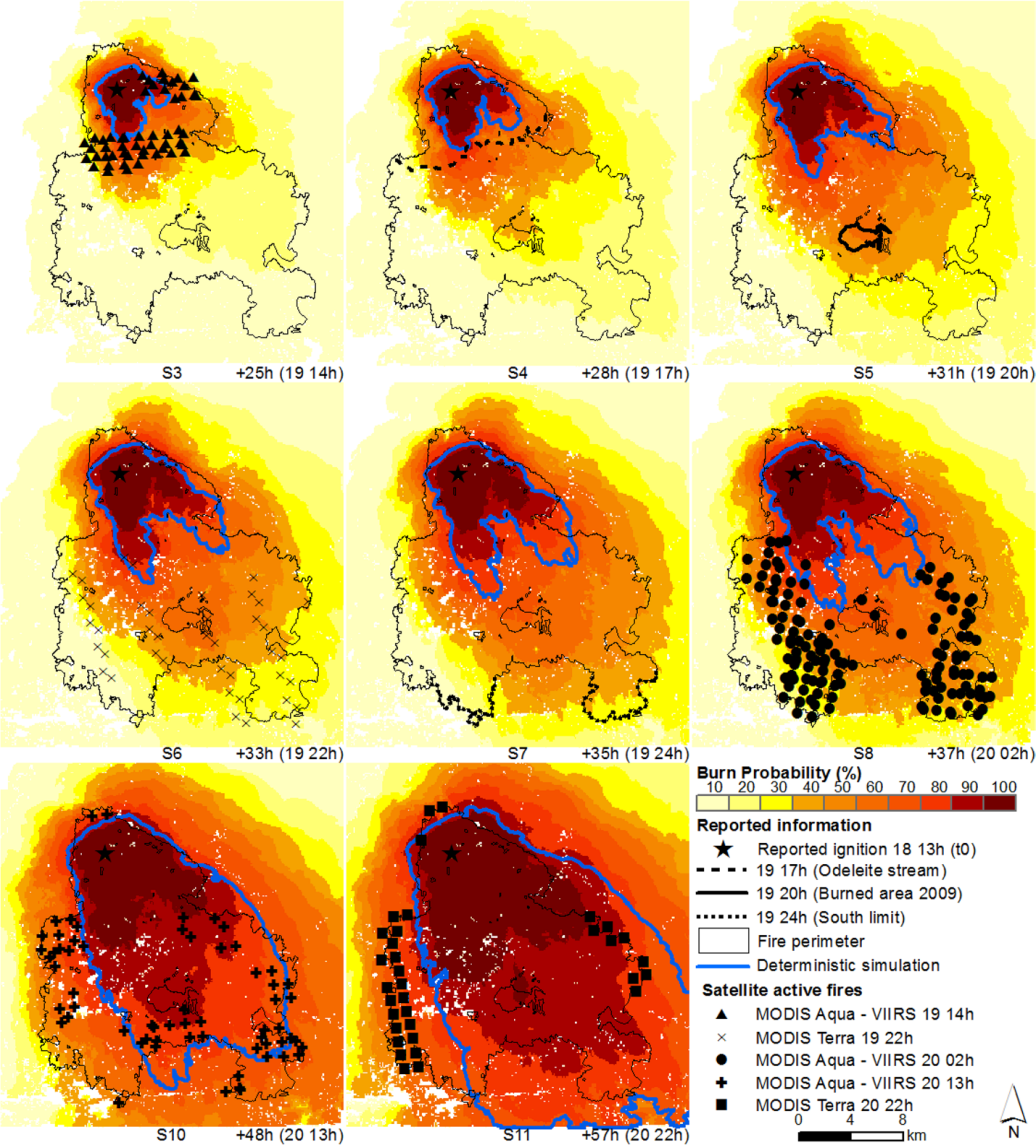
Probabilistic predictions (*shaded colours*) and deterministic simulations (*blue line*) of fire spread for the Tavira wildfire—t_0_. Simulations initialized at the reported ignition point (start: day 18 at 13 h; durations: 25 (S3), 28 (S4), 31 (S5), 33 (S6), 35 (S7), 37 (S8), 48 (S10) and 57 (S11) hours, respectively) and fire front location at the specified hour (time step), obtained from both satellite and reported information

Simulations were initialized at the reported ignition point, day 18 at 13 h (t_0_) and deterministic and probabilistic predictions of fire spread were produced for the Tavira wildfire (Fig. 7). The Tavira wildfire spread mainly towards the southeast, with overall wind direction from northwest (ANPC 2012; Viegas et al. 2012), which is reproduced in the fire spread simulations. However, higher probabilities and deterministic simulations show an under-prediction of fire spread when we compare each simulation with the location of the fire front at the different time steps (obtained from both satellite and reported information), in all sets of probabilistic predictions of fire growth representative of the most active period of the fire (until day 20 at 2 h, simulations 3–8). Cruz and Alexander (2013) examined a set of 49 fire spread model evaluation datasets and identified a significant underprediction regarding the fire’s surface rate of spread. Moreover, spotting contributed to fast fire growth during both phases (section “Case study: background and description”; ANPC 2012; Viegas et al. 2012), which was not simulated in this study in order to eliminate one possible confounding source of uncertainty, as spotting is stochastic in FARSITE. Spotting can advance fire many kilometres ahead of the main front and introduce substantial changes in fire spread pattern and behaviour (Finney 2004).

The advantage of probabilistic over deterministic simulations is clear when both are compared, since the former always overlap the fire front location at each time step, providing information regarding the probability of the fire to reach a certain location within a designated time. After the very fast spread towards south (until day 20 at 2 h), the fire spread towards the eastern and western boundaries of the fire perimeter (Fig. 5a, see the MODIS Aqua—VIIRS active fires day 20 at 2 h; and Fig. 5b, see the MODIS Terra and MODIS Aqua—VIIRS actives fires day 20 at 11, 13 and 22 h). The fire growth towards the west flank was underestimated by the deterministic simulations on day 20 at 2 h onward (Fig. 7, simulations 10 and 11) and did not reached this flank, while probabilistic predictions show probabilities equal or higher to 60 % of the fire reaching that flank, indicating a likely or probable occurrence (Cruz 2010).

### Probabilistic predictions of fire spread re-initialized with satellite active fire data

**Fig. 8:**
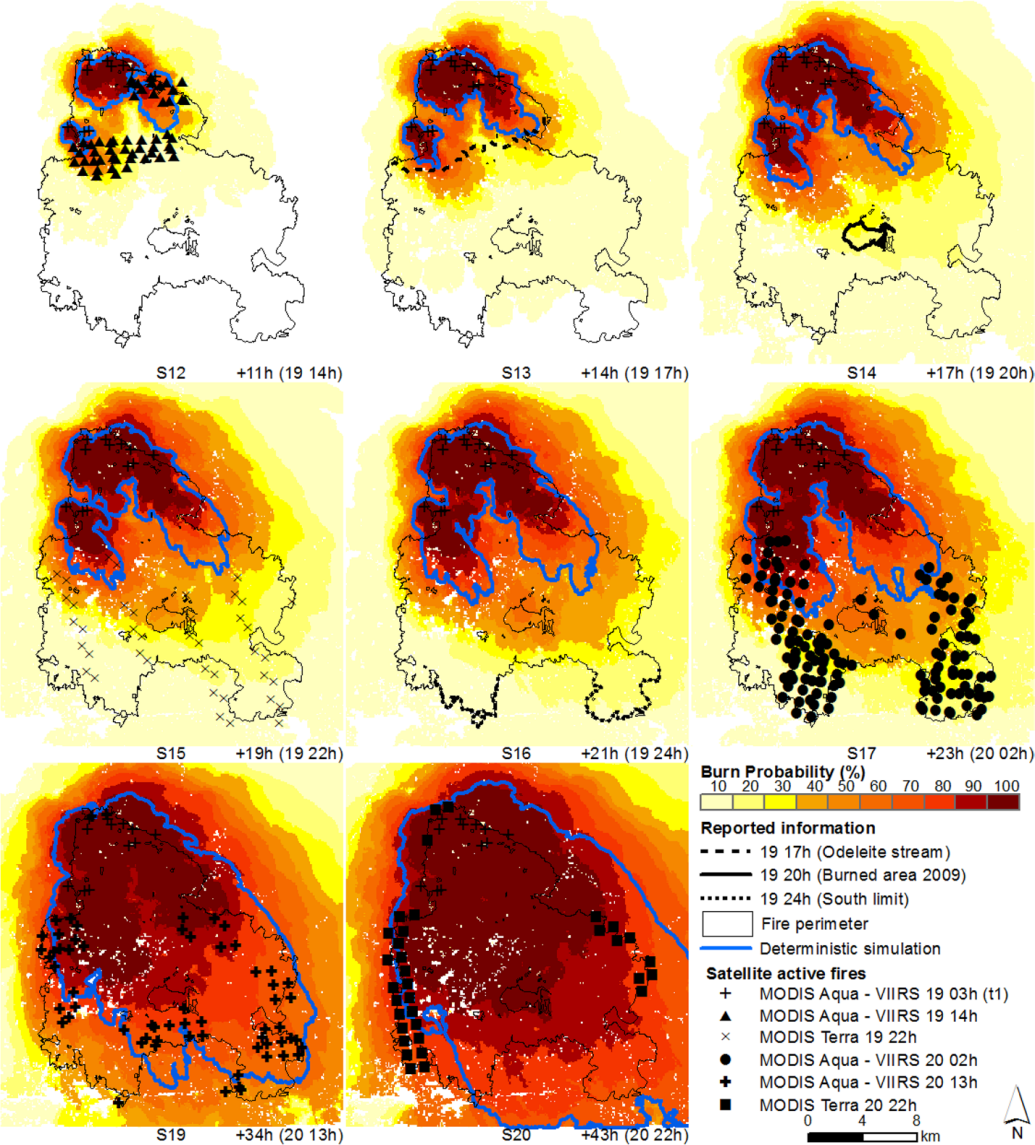
Probabilistic predictions (*shaded colours*) and deterministic simulations (*blue line*) of fire spread for the Tavira wildfire—t_1_. Simulations re-initialized with satellite active fires ignition points from the MODIS Aqua—VIIRS overpasses (start: day 19 at 3 h; durations: 11 (S12), 14 (S13), 17 (S14), 19 (S15), 21 (S16), 23 (S17), 34 (S19) and 43 (S20) hours, respectively) and fire front location at the specified hour (time step), obtained from both satellite and reported information

**Fig. 9:**
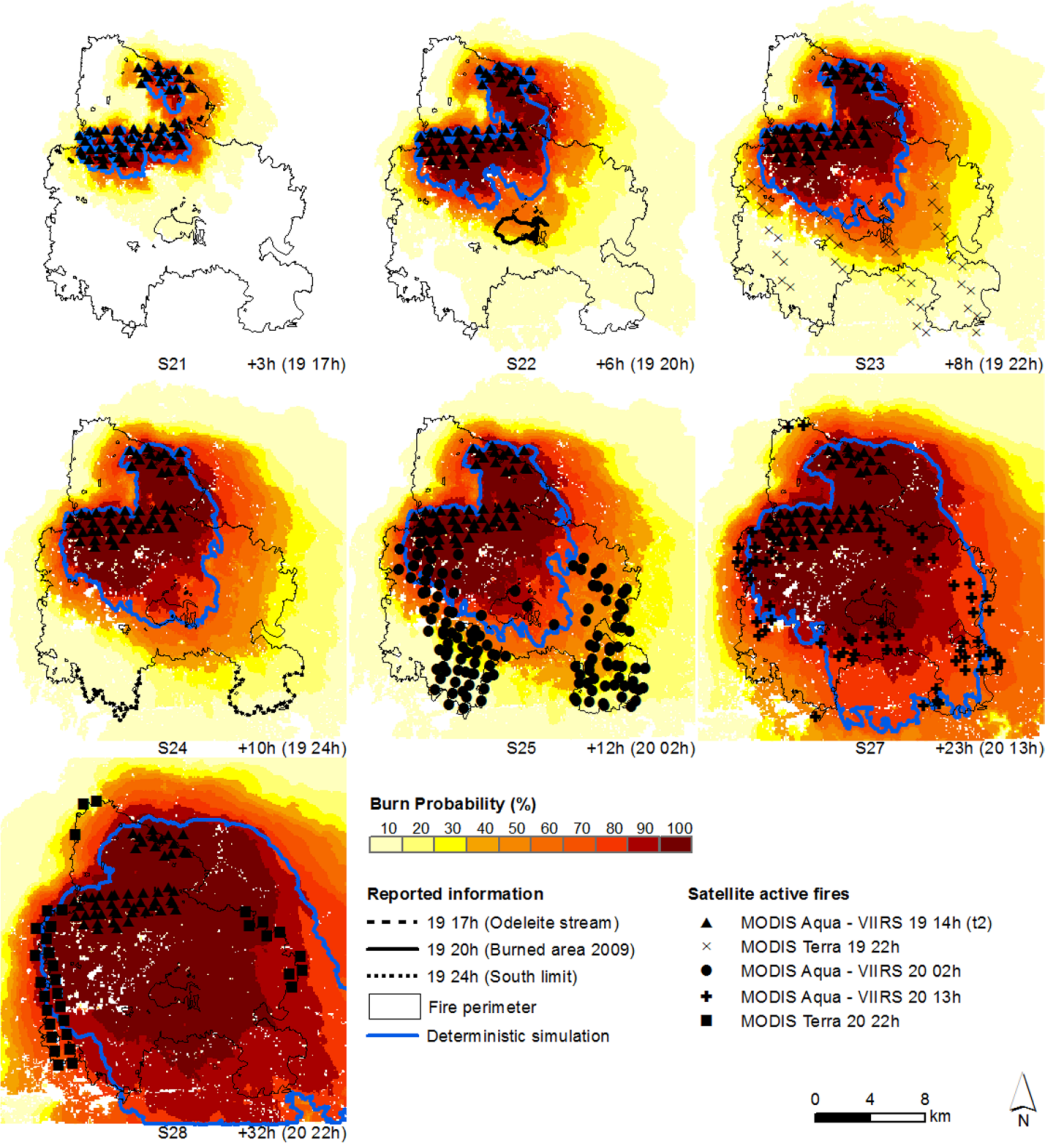
Probabilistic predictions (*shaded colours*) and deterministic simulations (*blue line*) of fire spread for the Tavira wildfire—t_2_. Simulations re-initialized with satellite active fires ignition points from the MODIS Aqua—VIIRS overpasses (start: day 19 at 14 h; durations: 3 (S21), 6 (S22), 8 (S23), 10 (S24), 12 (S25), 23 (S27) and 32 (S28) hours, respectively) and fire front location at the specified hour (time step), obtained from both satellite and reported information

Simulations were re-initialized with active fires from combined overpasses of both MODIS Aqua and VIIRS satellites, day 19 at 3 h (t_1_), day 19 at 14 h (t_2_) and day 20 at 2 h (t_3_) (Fig. 5a, section “Probabilistic fire spread simulations: framework and assessment”) and new deterministic and probabilistic predictions of fire spread were produced for the Tavira wildfire (Figs. 7, 8, 9, respectively).

**Fig. 10:**
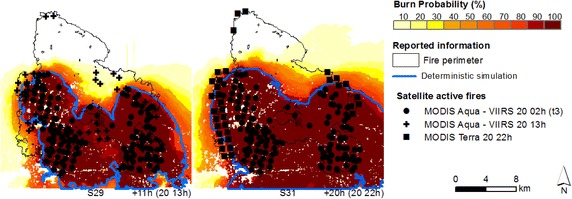
Probabilistic predictions (*shaded colours*) and deterministic simulations (*blue line*) of fire spread for the Tavira wildfire—t_3_. Simulations initialized with satellite active fires ignition points from the MODIS Aqua—VIIRS overpasses (start: day 20 at 2 h; durantion: 11 (S29) and 20 (S31) hours, respectively) and fire front location at the specified hour (time step), obtained from both satellite and reported information

For the period spanning between day 19 at 14 h and day 20 at 2 h, which comprises the most active period of the fire, when we compare the simulations initialized at t_0_ with the simulations re-initialized at t_1_ and t_2_, for the same time step (e.g. comparing simulations S6, S15, and S23, day 19 at 22 h, Figs. 7, 8 and 9 respectively), we observe that re-initializing simulations with satellite active fires did not improve simulations as expected, since fire spread continued to be underestimated, despite the spatial and temporal update of the fire front. Again, fire spread towards the west flank was underestimated by the deterministic simulations, from day 20 at 2 h onward (Fig. 8, simulations 19 and 20; Fig. 9, simulations 27 and 28; and Fig. 10, simulation 29).

**Fig. 11:**
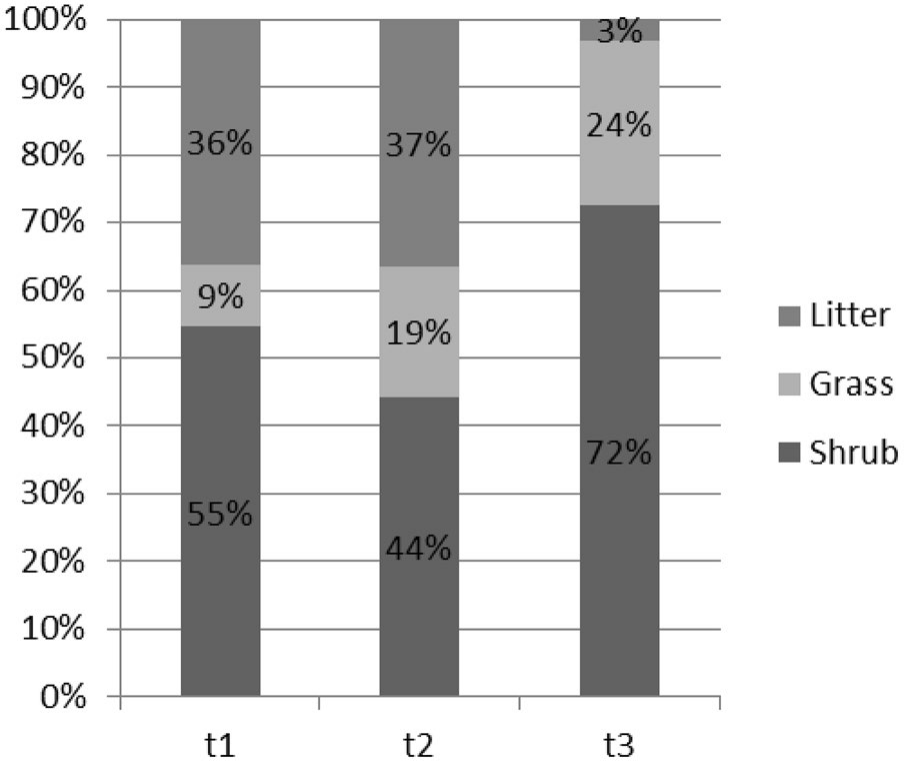
Satellite active fires frequency distribution over the landscape main fuel types (at t_1_, t_2_ and t_3_)

We explored the fuel type at the different ignition locations, at the start of the simulations. Figure 11 shows the percentage of active fires over each fuel type, for simulations re-initialized at t_1_, t_2_ and t_3_. For simulations initialized at t_0_ (single point) the fire started spreading through grass (FM2), whereas the active fires from simulations re-initialized at t_1_, t_2_ and t_3_ began spreading mainly through shrub (FM6) and litter (FM9) in the case of t_1_ and t_2_.

Although for simulations re-initialized at t_1_ and t_2_ most of the ignition points were located over shrubland, a considerable percentage of active fires were located over forest understory litter. Both grass and shrub fuel models have moderate-to-high rates of spread, while litter has low-to-moderate spread rates, and the ignitions occurring over this fuel type may have further delayed simulations (re-initialized at t_1_ e t_2_) comparing with simulations initialized at t_0_, which already had an active fire front when the fire reached this fuel type. Simulations at t_1_ and t_2_ were re-initialized as individual ignition sources rather than a fire line, and fire acceleration from a point source fire is slower than from a fire line (Finney 2004). In this regard, the use of active fires as points to re-initialize simulations must be further investigated, and additional research is needed to improve the assimilation of satellite active fire data.

**Fig. 12:**
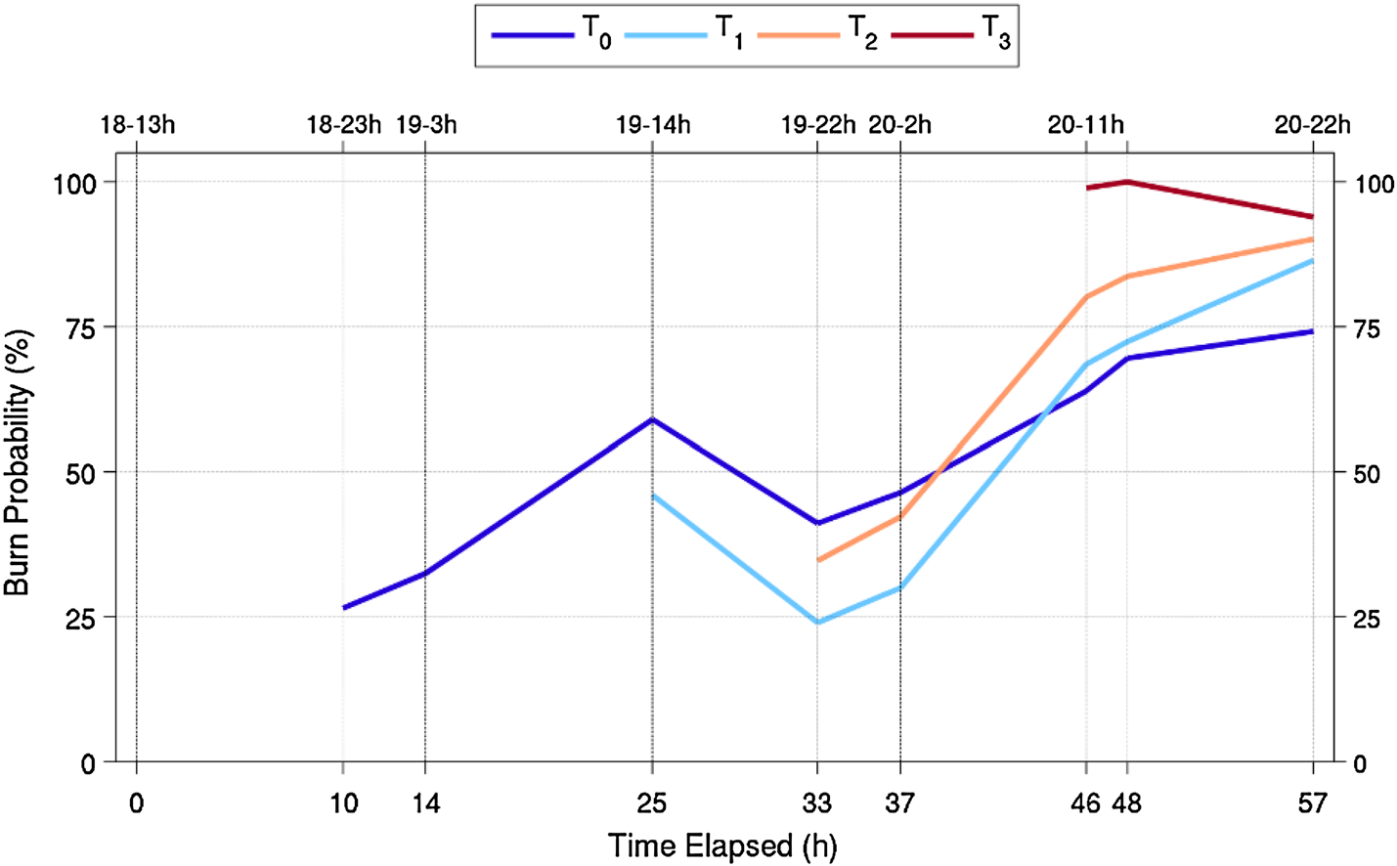
Median burn probability at the fire front location at the specified hour (time step), for simulations initialized at t_0_ and re-initialized at t_1_, t_2_ and t_3_. The *vertical lines* represent the day-time of ignition

Figure 12 shows the median burn probability within the active fires footprints, for simulations initialized at t_0_, t_1_, t_2_ and t_3_. A more pronounced fire spread under-prediction in simulations can be observed through the burn probability decrease, day 19 between 14 and 22 h. It is worthy to mention that during this period spotting occurred over 1500 m distances, causing new spot fires (spotting was not simulated). As previously stated, from day 19 at 14 h until day 20 at 2 h, initializing simulations at t_0_ produces fire spread predictions with higher burn probabilities than re-initializing simulations with satellite active fires at t_1_ and t_2_ (e.g. for day 19 at 22 h—t_0_, t_1_, and t_2_ with medium probability values of 40, 25 and 35 %, respectively). Nonetheless, t_2_ presents higher burn probabilities than t_1_ for this period. Given that the active fires are similarly distributed by fuel type, this difference may be due to the higher number of active fires in t_2_ (t_1_ n = 12; t_2_ n = 25) with larger sampling of the landscape and greater probability of encountering fuels and wind conditions favourable to faster growth (Finney 2000).

Simulations re-initialized at t_3_ present the highest burn probabilities within the active fires footprint, possibly due to a combination of factors: 72 % of the ignitions occurred over shrub fuel type (Fig. 11), a larger number of ignition points (n = 133) and a shorter simulation duration, with lower error accumulation. Later in the simulation’s time span, re-initializing simulations with satellite data produced better results comparing with t_0_, e.g., day 20 at 22 h, simulations t_0_, t_1_, t_2_ and t_3_ present medium probability values of 74, 86, 90 and 94 %, respectively. The underestimation of fire spread simulations initialized at t_0_ is now being counterbalanced by the re-initialization of simulations with satellite active fires. Nevertheless, likely or probable burn probabilities were still obtained for simulations initialized at t_0_ at this time.

### Potential of probabilistic predictions of fire spread as a decision support tool

The Tavira wildfire was a large and rapidly spreading fire, given its complex initial development and conflagration, which was beyond the existing fire suppression capabilities (section “Case study: background and description”). On the days of 20 and 21, less severe meteorological conditions coupled with better fire suppression conditions allowed for efficient fire containment.

One of the main problems encountered during the course of the fire-fighting operations was the poor information on the location and behaviour of the fire front during several phases of the fire, making it difficult to outline fire suppression strategies and leading to an insufficient assessment of fire potential (Viegas et al. 2012). Fire suppression decisions prioritize the protection of lives and assets, thus information regarding the fire’s behaviour, development and location during wildfire events tends to be incomplete (Anderson et al. 2009). Using satellite active fire data, the spatial–temporal dynamics of fire events can be reconstructed and relevant parameters regarding fire suppression, such as fire spread direction, rate of spread and fire intensity can be extracted (Parks 2014; Veraverbeke et al. 2014). This stresses the importance of using satellite data to monitor large wildfires and how combining different sources of information can provide a richer and more complete overview of their wildfire dynamics.

Although both MODIS and VIIRS satellites have a good visual agreement regarding the spatial–temporal distribution of active fires, they show inconsistencies with the reported information during the Tavira wildfire. Both reports (ANPC 2012; Viegas et al. 2012) state that the fire reached Odeleite Stream in day 19 at 17 h, 3 h later it reached the burnt area from 2009 (day 19 at 20 h) and 7 h later the fire reached its south limit (day 19 at 24 h) (Fig. 5b, section “Probabilistic fire spread simulations: framework and assessment”). However, both MODIS and VIIRS satellites detected active fires with different spatial and temporal distributions, e.g., both MODIS Aqua—VIIRS overpasses detected active fires in the Odeleite Stream day 19 at 14 h, earlier than reported by Viegas et al. (2012). In addition, the MODIS Terra overpass from day 19 at 22 h detected active fires beyond the area burned in 2009, close to the southern edges of the fire scar perimeter.

Combining fire spread modelling with satellite active fires did not always improve fire spread predictions through updating the fire front position with the active fires, when compared to simulations initialized at the reported ignition point. Fire spread is still under-predicted for the most active period of the fire (until day 20 at 2 h). However, the fire spread probability maps have a great potential to be used as a decision-support tool, integrating the uncertainty associated with input data and model predictions and providing information regarding the likelihood of fire growth across the landscape. For example, simulations in Fig. 7 show a medium probability of the fire reaching the active fire front location (above 40 % burn probability in most cases), at the specified time steps. This information was not obtained with the deterministic simulations.

During the event, the decision to allocate resources south of Odeleite Stream (on the afternoon of day 19) was based on the assumption that the fire was essentially contained. The underestimation of true fire potential led to a delayed response and resource allocation could have been reinforced to assure fire suppression during day 19 (Viegas et al. 2012). By anticipating the likelihood of fire spread further south of the Odeleite Stream and its major growth periods, the information given by the probabilistic predictions could have been useful in the operational setting regarding the decision-making process of fire suppression and pre-suppression activities at Odeleite Stream.

After day 20 at 2 h fire spread decreases, moving towards the eastern and western boundaries, as shown in Fig. 5 (Section “Probabilistic fire spread simulations: framework and assessment”), comparing fire progression between day 20 at 2 h (MODIS Aqua active fires, Fig. 5a) and day 20 at 11, 13 and 22 h (MODIS/VIIRS active fires, Fig. 5b). This change in fire progression was probably due to the combined effect of less severe meteorological conditions, characterized by higher relative humidity values and lower temperature values (ANPC 2012; Viegas et al. 2012) that led to more effective fire suppression. The deterministic simulations underestimated fire growth towards the west flank (Fig. 7, simulations 10 and 11; Fig. 8, simulations 19 and 20; Fig. 9, simulations 27 and 28; and Fig. 10, simulation 29). This section of the fire perimeter was intervened with bulldozers at the time of the event (Table 1), since the contiguous municipality presented great potential for fire spread (Viegas et al. 2012). The probabilistic simulations show medium to likely probability of the fire spreading beyond the flank, while the deterministic simulations did not reached this flank.

### Limitations and future work

The use of satellite active fire data to re-initialize simulations presents some issues, namely concerning pixel size variability and the uncertainty of sub-pixel fire front location (Anderson et al. 2009). The availability of new satellite active fire data products such as the new VIIRS 375 m (Schroeder et al. 2014) and Sentinel 3 will also contribute to provide additional information on active fires, with higher detection capabilities (Wooster et al. 2012). The sub-pixel location uncertainty assessment can also be improved, e.g., by using the sensor Point Spread Function (Campagnolo and Montano 2014). Multi-sensor approaches also allow for best temporal and spatial resolutions (Freeborn et al. 2009).

Fuel model uncertainty was not addressed in this work. A close look at the fuel model map produced by the municipalities (Fig. 1, section “Case study: background and description”) denotes a degree of subjectivity associated with fuel model classification by experts, which is clearly observed at the boundaries of both municipalities (between FM6 and FM7). Often, vegetation type maps such as the Corine Land Cover are used as base maps to assign fuel models, without a proper local model calibration and misinterpretation of fuel types can lead to erroneous estimates of spread rates and other fire behaviour properties (e.g. Arca et al. 2007; Anderson et al. 2009; Salis et al. 2013). Additionally, future work must include the fuel models most representative of shrubland and forest types for mainland Portugal (Fernandes et al. 2009).

We acknowledge that there are limitations with the spatial resolution of the meteorological data, however, the weather forecasts made available by IPMA and provided to ANPC during fire operational settings have the same resolution as the data used in this study, with a 72 h forecast range. Ultimately, this is the first readily available data to use in forecast mode, approximately 4 h after each model (ALADIN) run.

Future work will benefit from a more comprehensive understanding of uncertainty arising in fire spread modelling (Thompson and Calkin 2011), particularly from input data (e.g. Mowrer 2000; Anderson et al. 2007; Hilton et al. 2015). Uncertainty assessment is being further investigated by Benali et al. (2016). Relevant fire descriptors such as fire spread direction, rate of spread and fire intensity can also be extracted from satellite data and compared to fire behaviour output parameters from FARSITE, for further assessment and identification of opportunity windows for more efficient fire suppression operations.

## Conclusions

The use of fire spread models as a decision-support tool for fire management must quantify and integrate the uncertainty associated with input data uncertainty and thus probabilistic approaches should be favoured over deterministic ones.

The fire spread probability maps produced, provide valuable and additional information regarding the spatial and temporal distribution of burn probabilities, such as where, when and with what probability the fire might be in the next few hours. This information allows anticipating fire spread through the landscape with an associated probability of occurrence, which combined with expert knowledge and judgment, presents great potential to be used as a decision-support tool for fire suppression and pre-suppression management, in operational settings. The proposed approach can be useful to forecast the growth of future wildfires, in particular large and infrequent wildfires.

## Additional file

10.1186/s40064-016-2842-9 Fuel models (Northern Forest Fire Laboratory, NFFL) of the study area. **Figure S2.** Topographic features of the study area. **Table S1.** Summary of the datasets used to derive the model input data.

## Abbreviations

ANPC: National Authority for Civil Protection
FARSITE: Fire Area Simulator
FM: Fuel Model
FWI: Fire Weather Index
MODIS: MODerate Resolution Imaging Spectroradiometer sensor
NFFL: Northern Forest Fire Laboratory
PDF: Probability Density Functions
TOA: time of arrival
ROS: rate of spread
VIIRS: visible infrared imaging radiometer suite
