# Supplementary material for: Probabilistic fire spread forecast as a management tool in an operational setting
Source: Springerplus. 2016 Jul 28;5(1):1205. doi: 10.1186/s40064-016-2842-9 (PMC4965362; doi:10.1186/s40064-016-2842-9)
Supplement: Supplementary file 1 — 10.1186/s40064-016-2842-9 Fuel models (Northern Forest Fire Laboratory, NFFL) of the study area. Figure S2. Topographic features of the study area. Table S1. Summary of the datasets used to derive the model input data. [file 40064_2016_2842_MOESM1_ESM.docx]

Additional file 1


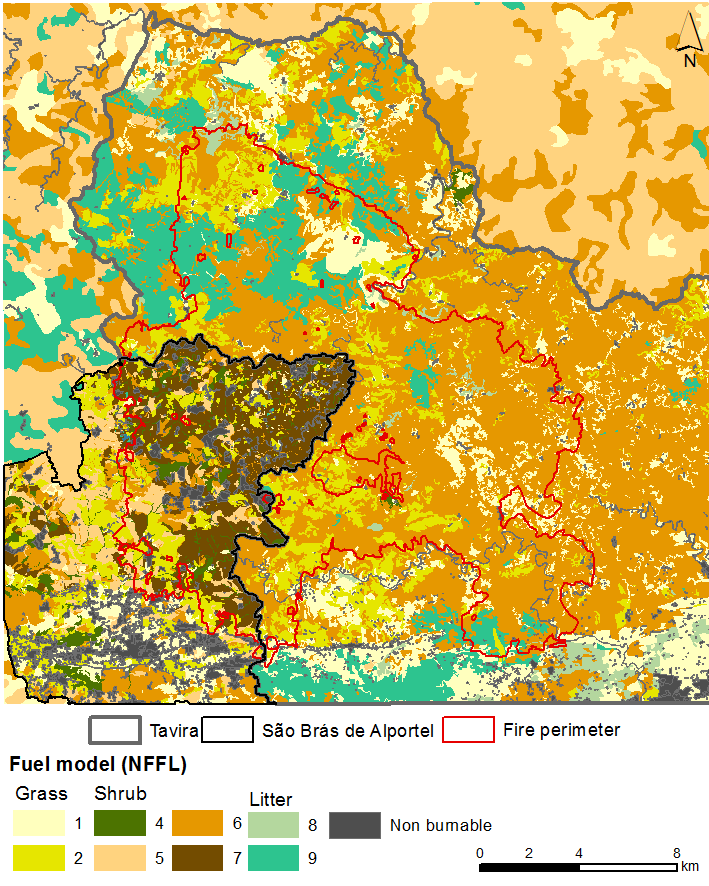


**Figure S1.** Fuel models (Northern Forest Fire Laboratory, NFFL) of the study area


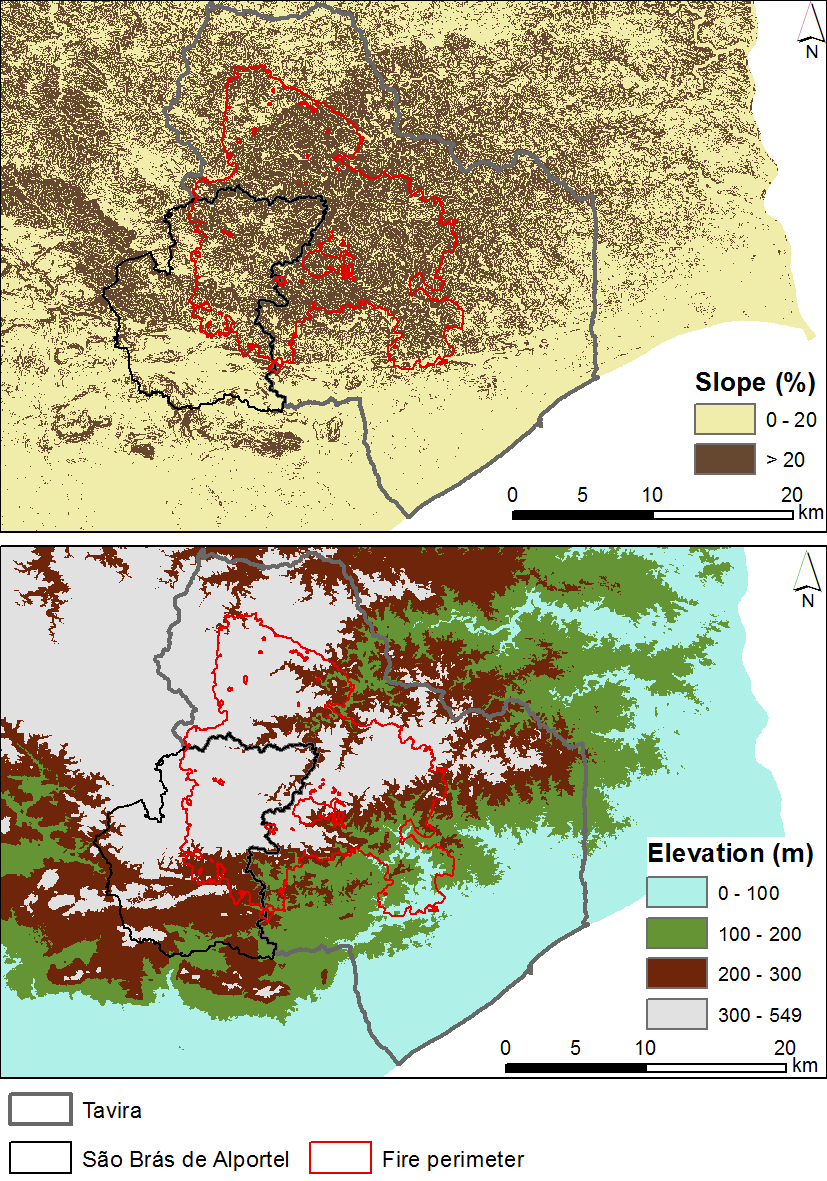


**Figure S2.** Topographic features of the study area

**Table S1.** Summary of the datasets used to derive the model input data

| **Data sets** | **Purpose/Input variables** | **Source** |
| --- | --- | --- |
| Digital Elevation Model provided by the Shuttle Radar Topography Mission (90 m) | Derive elevation, slope and aspect to produce the topography maps. Raster data aggregated to 100 m cell size. | https://lta.cr.usgs.gov/SRTM |
| Fuel maps adopting the Northern Forest Fire Laboratory 13 standard fire behaviour fuel models classification | Derive fuel model maps. Raster data with 100 m cell size. | Produced by the Portuguese municipalities affected by the wildfire |
| MODIS Vegetation Continuous Fields Yearly L3 Global (MOD44B) (250m) | Derive canopy cover. Raster data resampled to 100 m cell size. | https://lpdaac.usgs.gov/dataset_discovery/modis/modis_products_table/mod44b |
| Portuguese National Forest Inventory (2005-2006) | Derive stand height, canopy base height and canopy bulk density maps. Raster data with 100 m cell size. | http://www.icnf.pt/portal |
| Wind speed and direction simulated by the Weather Research and Forecast model (9km) | Gridded wind data further modelled with WindNinja to account for the effect of the interaction between topography and local winds. Output raster data with 100 m cell size. | Provided by the Climatology and Climate Change group at the Institute Dom Luis, Faculty of Sciences, University of Lisbon |
| Meteorological variables simulated by the WRF model (9km) | Create weather data streams summarized on a daily basis. | Provided by the Climatology and Climate Change group at the Institute Dom Luis, Faculty of Sciences, University of Lisbon |
| MODIS active fire product (MCD14ML) (1 km) | Derive ignition points. The centroid coordinate was assumed and the closest 100 m grid cell was assigned. | http://modis-fire.umd.edu/pages/ActiveFire.php |
| VIIRS active fire product (750m) | Derive ignition points. The centroid coordinate was assumed and the closest 100 m grid cell was assigned. | http://viirsfire.geog.umd.edu/ |
